# Emergency Front-of-neck Access: Comparing Needle and Surgical Cricothyrotomy Using a Human Cadaver Model

**DOI:** 10.5811/westjem.52840

**Published:** 2026-06-28

**Authors:** Eric Boccio, Daniel Levi, James Bonz, Justin Belsky, Jason D’Amore

**Affiliations:** * Mount Sinai Medical Center of Florida, Department of Emergency Medicine, Miami Beach, Florida; † Baptist Health, Department of Emergency Medicine, Miami, Florida; ‡ Yale New Haven Hospital, Department of Emergency Medicine, New Haven, Connecticut; § Health + Hospitals/Jacobi Bronx, Department of Emergency Medicine, New York City, New York

## Abstract

**Introduction:**

Emergency front-of-neck access may serve as a life-saving intervention when facing patients with difficult airways. Cricothyrotomy consists of needle and surgical techniques and is performed in approximately 0.2–0.5% of all airway management attempts. Our primary aim in this study was to compare procedural completion time and first-pass success of needle and surgical approaches to cricothyrotomy by civilian and military practitioners using a human cadaver model.

**Methods:**

Emergency medicine (EM) attendings and residents and U.S. Air Force Pararescue specialists were randomized to perform either a needle or surgical cricothyrotomy on an unfixed human cadaver a single time following a 30-minute didactic session about both procedure types. We recorded procedural completion time, first-pass success, frequency and type of observed complications, and subject level of training. Our primary outcome measure was procedural completion time. Secondary outcome measures included first-pass success and complication rates. We used a Wilcoxon signed-rank test to compare the difference in median completion times between needle and surgical groups. We performed a Cox regression analysis to evaluate the relationship between technique and procedural completion time while adjusting for subject level of training. Chi-squared or Fisher exact tests were used to compare unadjusted first-pass success and complication frequencies between needle and surgical groups. Multivariable logistic regression analysis modeling the association between technique and first-pass success and complication events while adjusting for subject level of training was performed.

**Results:**

A total of 99 subjects were enrolled, and 19 (19%), 68 (69%), and 12 (12%) were classified as EM attending, EM resident, and Pararescue specialist, respectively; 51 (52%) and 48 (48%) were randomized into the needle and surgical groups, respectively. The median time to procedural completion was shorter in the needle group than the surgical group (56.5 seconds [sec], 95% confidence interval, 54–66 sec vs 65 sec, 95% CI, 59–73 sec, respectively). The difference in median completion times was 8 sec (95% CI, −1.0 to 17 sec, P = .08). The hazard ratio comparing completion time of surgical to needle cricothyrotomy while adjusting for level of training was 1.41 (95% CI, 0.93–2.13, P = .11). First-pass success and complication rates were similar between the needle and surgical groups (94% vs 94%, P = .32, and 27% vs 33%, P = .52, respectively). The adjusted odds ratios comparing the likelihoods of first-pass success and complication between surgical and needle groups while adjusting for level of training were 1.06 (95% CI, 0.20–5.54, P = .95) and 1.40 (95% CI, 0.55–3.56, P = .48), respectively.

**Conclusion:**

In this study using unfixed human cadavers, needle and surgical cricothyrotomy demonstrated comparable performance regarding procedural completion time, first-pass success, and complication rates. The 8-second difference in median completion time between groups was not found to be statistically significant and is unlikely to be clinically significant given the typical oxygen reserves in an apneic patient. These findings suggest that for practitioners in civilian and military settings, both needle and surgical cricothyrotomy remain viable options for emergency front-of-neck access, assuming adequate operator-level procedural proficiency and access to necessary equipment.

## INTRODUCTION

Emergency front-of-neck access may serve as a life-saving intervention when facing the “cannot intubate, cannot oxygenate” airway. Cricothyrotomy is performed in approximately 0.28% of all airway management attempts in the emergency department (ED),^1^ and similar rates (0.5%) have been reported in the prehospital setting.^2^ There are two common classifications of cricothyrotomy approaches: needle/percutaneous/Seldinger (needle); and surgical/open (surgical).^3^ The relatively high acuity and low occurrence of difficult airways limit opportunities for performing cricothyrotomy and maintaining procedural proficiency through clinical practice alone.^4^ There is a paucity of research comparing procedural performance of needle and surgical cricothyrotomy by emergency physicians and military medical personnel.

Previous studies have been largely limited to small samples of anesthesiologists and medical students observed performing emergency front-of-neck access on simulated airway models. As a result, strong evidence to support superiority of either approach is lacking. In clinical scenarios where there is a critical need to access, secure, oxygenate, and ventilate the airway as quickly as possible, the approach that can be performed faster, with a higher success rate, and which minimizes complications should be prioritized. This study builds upon the existing evidence by pairing a human cadaveric model, which preserves realistic anatomical landmarks, with a targeted cohort of emergency physicians and prehospital military personnel in a high-fidelity simulated environment.

Our primary aim in this study was to determine whether needle or surgical cricothyrotomy could be completed faster by practitioners responsible for performing emergency front-of-neck access in the civilian and military settings, using a human cadaver model. Secondary outcomes focused on comparing first-pass success and procedural complication rates.

## METHODS

### Study Design and Setting

To compare cricothyrotomy techniques, we performed a randomized controlled trial using a human cadaver model in a simulated environment. We recruited emergency physicians from a single, urban, academic institution in the northeast United States with an annual census of approximately 100,000 adult patients and an affiliate Accreditation Council for Graduate Medical Education (ACGME)-accredited EM residency program. At the time of the study, equipment across all clinical sites was standardized and featured the Melker Universal Emergency Cricothyrotomy Catheter Set (Cook Medical Inc, Bloomington, IN), which contained the necessary equipment to perform both needle and surgical techniques. There was no single standardized approach to emergency front-of-neck access practiced and residents were instructed on both. The study protocol was reviewed and approved by a central institutional review board prior to subject enrollment.

**Population Health Research Capsule**

What do we already know about this issue?

*Emergency front-of-neck access is a rare, life-saving airway intervention, but evidence comparing needle and surgical techniques is limited by small samples*.

What was the research question?

*Does needle or surgical cricothyrotomy have faster completion time, higher rate of success, and fewer complications?*

What was the major finding of the study?

*Needle and surgical cricothyrotomy had similar median completion times (56.5 vs 65 seconds, hazard ratio 1.41), first-pass success (94%), and complication rates (27% vs 33%)*.

How does this improve population health?

*Both techniques are viable options, allowing providers to prioritize the method that best matches their proficiency and available equipment*.

### Selection of Participants

Subjects were selected from a stratified random sample of participants attending a one-day advanced airway management training course held in January 2017. Subjects consisted of EM attendings and residents and U.S. Air Force Pararescue specialists, and the sample was stratified based on type and level of training. In addition to basic military, combat dive, airborne, and survival, evasion, resistance, and escape training, Pararescue specialists may serve as licensed emergency medical technicians, paramedics, and field/combat medics and are trained and certified to perform emergency front-of-neck access in both civilian and military prehospital environments.

### Interventions

Unfixed, fresh-frozen human cadavers were numbered sequentially, and subjects were randomly assigned to a cadaver. The cadavers were fresh-frozen to maintain tissue, color, and texture. Subjects assigned to odd-numbered cadavers were instructed to perform the needle technique, while those assigned to even-numbered cadavers were instructed to perform the surgical technique. Each subject was instructed to perform the respective cricothyrotomy technique a single time, and each cadaver underwent cricothyrotomy a single time. All subjects were given a complete Melker set and advised to use the equipment necessary to perform their assigned approach. A single subject failed to follow instructions and performed a needle cricothyrotomy instead of surgical cricothyrotomy. Data pertaining to this subject were classified in the needle cricothyrotomy group during analysis. The cadavers were fresh-frozen to maintain tissue, color, and texture. All subjects were given a complete Melker set and advised to use the equipment necessary to perform their assigned approach.

Immediately preceding each attempt, subjects received a 30-minute didactics session taught by a board-certified emergency physician (JD) on emergency front-of-neck access, which featured content related to needle and surgical approaches to cricothyrotomy. For the needle approach, a Seldinger catheter-over-wire technique was described. After anatomic landmarks were identified and stabilized with the non-dominant hand, the introducer needle attached to a syringe containing 2 mL saline was inserted through the skin and subcutaneous tissue overlying the cricothyroid membrane caudally at a 45° angle.

The plunger of the syringe was simultaneously drawn back as the needle advanced until air was aspirated. Entrance into the trachea was confirmed through visualization of bubbles in the syringe. The syringe was subsequently removed, and a guide wire was inserted and passed through the needle shaft. While stabilizing the guide wire, the needle was then removed. A stab incision was made in the skin at the guide wire insertion site, and a dilator was advanced over the guide wire. The dilator was exchanged for a cannula, which was passed over the guide wire. Once properly positioned, the cannula was stabilized, and the guide wire was removed.

For the surgical approach, a scalpel-finger-endotracheal tube technique was described. Anatomic landmarks were first defined by thyroid cartilage superiorly, cricothyroid muscles laterally, and cricoid cartilage inferiorly. While stabilizing the larynx with the non-dominant hand, a cutting scalpel was used to create a vertical incision through the skin and subcutaneous tissue overlying the cricothyroid membrane. Once identified via direct visualization or palpation, a horizontal incision was made through the lower portion of the cricothyroid membrane. The index finger of the non-dominant hand was inserted through the stoma thus maintaining the tract and confirming proper positioning via palpation of the posterior tracheal rings. A 6.0 endotracheal tube was inserted, and the cuff was fully inflated. The use of a tracheal hook and gum elastic bougie was not described and, therefore, not investigated.

### Outcome Measures

We defined the primary outcome measure—procedural completion time—as a continuous variable. Secondary outcome measures included first-pass success and observed complications, which were defined as binary variables. We described and summarized observed equipment and procedural complications to comment on the general safety of each technique. Complication types were discussed via group expert consensus (EB, JB, JB, JD) a priori and classified as follows: 1) missed or repeated step; 2) needle insertion or incision not midline; 3) structural damage to the cricoid or thyroid cartilage; 4) structural damage to the trachea; and 5) ectopic catheter or endotracheal tube placement.

### Data Collection

We recorded procedural completion time, first-pass success, frequency and type of observed complications, and subject’s level of training. Procedural completion time, in seconds (sec), was measured on digital stopwatches by designated research personnel (EB, JD) who received training on proper timing of the procedure. This was defined as the interval between initial skin contact with the needle tip or scalpel blade and the insertion of the catheter or endotracheal tube tip into the neck stoma, as directly visualized by research personnel at the bedside in real time. After each attempt, further dissection of the cadaveric tissue by research personnel was performed, and successful placement was confirmed through direct visualization of the catheter or endotracheal tube passing through the cricothyroid membrane and terminating in the trachea. It was also noted whether insertion and incision sites were midline, as injury to the perforating branches of the thyroid artery may be associated with greater bleeding complications.

### Sample-size Calculation

Assuming mean completion times of 148 (96) sec and 55 (35) sec for first-pass success while using a percutaneous and surgical approach, respectively (as reported in a previous study of prehospital personnel performing cricothyrotomies on human cadavers^5^), 90% statistical power, and a two-sided alpha of 0.05, we needed a sample of 44 subjects to confidently detect a difference between procedural completion times between the two study arms. On the day of subject enrollment 99 unfixed human cadavers were available, and all were used.

### Statistical Analysis

We calculated summary statistics for each group and each outcome. A Wilcoxon signed-rank test was used to compare unadjusted median completion times between needle and surgical groups. We used chi-squared or Fisher exact tests to compare unadjusted first-pass success and complication frequencies between needle and surgical groups. Kaplan-Meier curves illustrating time to procedural completion for each technique for the overall sample and stratified by subject level of training were created. We performed a Cox regression analysis to evaluate the relationship between technique and procedural completion time while adjusting for subject level of training. The ratio of hazard rates between the two groups was assumed to remain constant over time. We performed multivariable logistic regression of first-pass success and complication events between techniques while adjusting for subject level of training. We assessed the goodness of fit for each model using a Hosmer-Lemeshow test. The threshold for statistical significance was set at 0.05 a priori. All analyses were performed using SAS Studio v9.4 (SAS Institute Inc, Cary, NC).

## RESULTS

### Characteristics of Study Subjects

Of the 99 subjects who were enrolled and included in analysis, 19 (19%), 68 (69%), and 12 (12%) were classified as EM attending, EM resident, and Pararescue specialist, respectively. A total of 51 (52%) and 48 (48%) subjects were randomized to the needle and surgical groups, respectively (Table 1). Of the cadavers characterized as obese based on subjective assessment of neck and torso size by research personnel (EB, JD), 12 (24%) and 5 (10%) were in the needle and surgical groups, respectively.

### Results of Median Time to Completion and Rates of Complication

The median time to procedural completion was shorter in the needle group as compared to the surgical group (56.5 sec, 95% CI, 54–66 sec vs 65 sec, 95% CI, 59–73 sec, respectively). The difference in medians was 8 sec (95% CI, −1.0 to 17 sec, *P* = .08). Three (6%) subjects in the needle group and three (6%) in the surgical group failed on first attempt. Complications observed in the needle group included missed or repeated procedural step (additional dilation attempt), structural damage to the cricoid or thyroid cartilage, and ectopic catheter placement. Complications observed in the surgical group included incision not midline, structural damage to the cricoid or thyroid cartilage, structural damage to the trachea, and ectopic endotracheal tube placement (Table 2).

**Figure 1:**
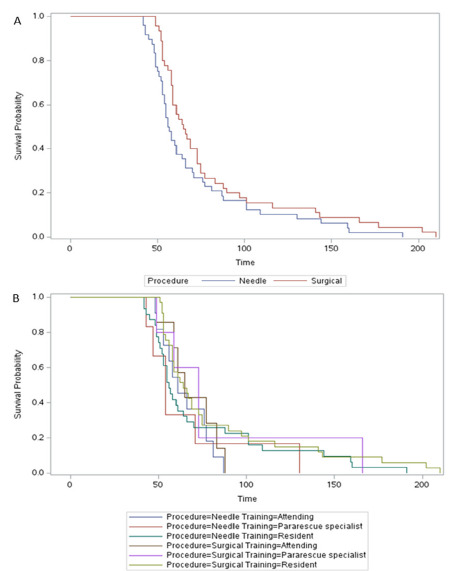
Kaplan-Meier curves illustrating (A) time to procedural completion of surgical and needle techniques and (B) stratification by subject level of training, in a study comparing procedural performance of cricothyrotomy on unfixed human cadavers.

The models for first-pass success and complication occurrence provided good fits to the data, as indicated by non-significant Hosmer-Lemeshow tests (*P* = .47 and *P* =.56, respectively). First-pass success and observed complication rates were similar between the needle and surgical groups (94% vs 94%, *P* = .32, and 27% vs 33%, *P* = .52, respectively). Kaplan-Meier curves illustrating time to procedural completion for each technique for the overall sample and stratified by subject level of training are displayed in Figure 1.

The hazard ratio comparing completion time of surgical cricothyrotomy to needle cricothyrotomy while adjusting for subject level of training was 1.41 (95% CI, 0.93–2.13, *P* = .11). The adjusted odds ratios comparing the likelihoods of first-pass success and complication between surgical and needle groups while adjusting for subject level of training were 1.06 (95% CI, 0.20–5.54, *P* = .95) and 1.40 (95% CI, 0.55–3.56, *P* = .48), respectively. Subject type and level of training were not found to be statistically significant in any of the regression models.

## DISCUSSION

In this human cadaveric study of cricothyrotomy techniques, the 8-second difference in median completion times between the needle and surgical groups was not found to be statistically significant by univariate analysis or Cox regression while adjusting for subject level of training. Comparison of the 75^th^ percentiles of completion times in the needle and surgical groups demonstrates only a minor difference, unlikely to be of clinical significance when considering the theoretical risks during such a brief extension of the apneic period. Comparison of the crude Kaplan-Meier curve demonstrates an earlier drop in the needle group representing faster rates of completion compared to the surgical group. When stratified based on subject level of training, the fastest 80% of attempts appear to have similar completion times across techniques. Variability in completion time was seen among the slowest 20% of attempts with EM attendings completing cricothyrotomy the fastest followed by Pararescue specialists and then EM residents. Emergency medicine attendings appeared to be equally proficient when performing either needle or surgical cricothyrotomy, while Pararescue specialists and EM residents were slower when performing the surgical technique.

Both needle and surgical groups had almost identical high first-pass success rates (~94%) with similar complication rates, and these differences were not found to be statistically significant. However, the types of complications observed differed between techniques. Missed or repeated procedural steps were only observed in the needle group, while incision not midline and damage to the cricoid or thyroid cartilage were only observed in the surgical group. Rates of damage to the trachea and incorrect positioning of equipment were similar between groups. The difference in observed complications between groups is most likely attributable to the micro skills composing each technique.

The needle technique requires more equipment and is composed of more individual steps than the surgical technique. Failure to adequately dilate is unique to the needle technique as the surgical technique does not involve the use of a dilator. The presence of bubbles in the saline-filled syringe during aspiration while performing the needle technique may provide visual feedback confirming entrance into the trachea. This likely explains why lateral insertion and damage to the cricoid or thyroid cartilage were observed only in the surgical group where subjects relied on anatomic landmarks and blind dissection through soft tissue to identify the cricothyroid membrane. Incisions made too deeply—laterally or above or below the cricothyroid membrane— were more likely to result in damage to cricoid or thyroid cartilage during the surgical technique. Although there were twice the number of obese cadavers in the needle group than the surgical group, we observed similar first-pass success and complication rates.

Cadaveric and simulation-based studies comparing emergency cricothyrotomy techniques among EM attendings, trainees, and other clinicians have shown broadly comparable outcomes, with variation in completion times, operator preference, and training influences. In one human cadaveric study, 20 emergency physicians performed 200 cricothyrotomies using both Seldinger and surgical methods. Although repetition did not improve proficiency, the Seldinger approach achieved shorter times to tracheal puncture and ventilation, with similar complication rates (11.8 vs 16%).^6^ A randomized crossover trial of 15 EM attendings and residents likewise found no significant differences in completion time, placement accuracy, or complication rates between wire-guided and surgical approaches. Success was slightly higher with the wire-guided method (93.3 vs 86.7%), and participants preferred Seldinger, although the clinical significance of smaller incision size and subjective ease remains unclear.^7^ A prospective, randomized, comparative simulation study using a human cadaver model involving 63 inexperienced clinicians showed similar median completion times between surgical and catheter-over-needle techniques (78 vs 74 sec, respectively), with modestly higher success in the surgical group (94% vs 82%). We attributed slower times and higher failure rates to participant inexperience, limiting generalizability.^8^ Similarly, a trial of 20 medical students naïve to surgical airways reported a 95% success rate with the surgical technique, although the median completion time was slower (94 sec) and one complication involved esophageal placement. No association was found between cadaver biometric features and outcomes, reinforcing the influence of subject level of training.^9^

Conversely, some trials favored the surgical technique. A crossover study of Australian emergency physicians and trainees demonstrated a faster mean completion time while performing the surgical compared to the Seldinger technique (51.6 [16.3] sec vs 66.6 [14.9] sec, respectively), possibly reflecting training differences internationally.^3^ Likewise, anesthesiologists comparing open surgical and Melker approaches found the surgical method faster and less technically difficult, with a difference in mean completion time of 42 sec. Despite slower completion times, participants preferred the Melker technique, likely due to familiarity with Seldinger-based micro skills, although generalizability to EM practice is limited.^10^

The National Association of Emergency Medical Services Physicians (NAEMSP) strongly supports open scalpel cricothyrotomy over needle-based or Seldinger techniques for emergency front-of-neck access in the prehospital setting. The most recent position statement cites previous work demonstrating markedly higher success rates and a 2% failure rate when performing surgical cricothyrotomy compared to 65% for needle-based methods. Advantages reported include reduced reliance on specialized equipment and the ability to use adjuncts such as a tracheal hook or gum elastic bougie to confirm tract position. Our results corroborate the concern that additional equipment and individual steps may lead to higher observed complication rates when performing the needle technique; however, we are unable to comment on their clinical significance.

Additionally, while the inclusion of a tracheal hook or a gum elastic bougie may mitigate ectopic endotracheal tube placement during the surgical technique, we did not investigate these adjuncts in our study. However, it should be noted that the visualization of bubbles while aspirating with saline in the syringe during the needle technique does provide feedback regarding proper positioning of the needle in the trachea prior to guidewire insertion. While procedural modifications like vertical incision may mitigate common pitfalls such as misidentification of cricoid landmarks and ectopic endotracheal tube placement, our results show that these complications were not eliminated completely. While the NAEMSP strongly supports an open surgical scalpel-based approach as first line to emergency front-of-neck access, we agree with the statement supporting regular simulation training in multiple emergency front-of-neck access techniques and further agree that additional controlled clinical trials of emergency surgical airways are necessary to strengthen current and future recommendations.^11^

Hybrid approaches to emergency front-of-neck access have also been evaluated. An “incision-first” Seldinger variation reduced procedural time, insertion attempts, and workload compared with a “needle-first” approach.^12^ A sequential technique combining transtracheal catheter oxygenation with Seldinger cricothyrotomy has likewise been proposed to expedite preoxygenation.^13^ Overall, evidence suggests both surgical and Seldinger methods are effective, with optimal technique influenced by operator experience, procedural familiarity, and available equipment.^14^

## LIMITATIONS

Subjects were recruited from those in attendance at an advanced airway management training course, which exposed our study to selection bias. While subjects did not receive hands-on training of needle or surgical cricothyrotomy techniques prior to their attempt, each subject participated in a didactic session that reviewed the procedural steps of each approach. Emergency physicians were representative of a single academic department while EM residents were representative of a single ACGME-accredited program, both located in the Northeast U.S. and thus limiting generalizability.

While the unfixed human cadavers were screened for obesity prior to each attempt, we did not control for other anatomical variations that may have affected the difficulty in performing each technique (neck length and circumference, history of head and neck cancer, prior trauma, etc). While considered higher fidelity than manikin trainers, unfixed cadaveric tissue does not allow for assessment of neck vasculature; neither could bleeding from vascular injury be simulated. The severity of patient injury from procedural complications could not be quantified and, therefore, is speculative. Additionally, rigor mortis may have prevented the ability to fully extend and position the neck prior to each attempt. The controlled environment of a cadaver lab cannot fully replicate the cognitive load, time pressure, and environmental distractions inherent in real-life clinical scenarios involving emergent advanced airway management.

Subject demographics and prior clinical experience related to total number of years of experience and number of cricothyrotomies performed were recognized as potential confounders, but this information was not recorded. The risk of potential confounding was mitigated through block randomization of the procedure. After stratification by subject level of training, strata containing Pararescue specialists have small sample sizes (*n* < 10). Results drawn from subgroup analyses are, therefore, believed to be underpowered, and results could vary if the study were to be replicated with larger sample sizes. Finally, tracheal hooks and gum elastic bougies are common adjuncts used when performing surgical cricothyrotomy; however, we did not assess their use.

## CONCLUSION

This human cadaveric-based study investigated the optimal approach to emergency front-of-neck access, addressing the limitations of prior studies through use of high-fidelity, unfixed human cadavers, randomization of subjects into procedural arms, and inclusion of a larger sample size of EM-trained subjects. The 8-second difference in median completion time between groups was not found to be statistically significant and is unlikely to be clinically significant given the typical oxygen reserves in an apneic patient. Both techniques achieved an identical 94% first-pass success rate with comparable overall complication rates, although the specific nature of complications differed based on the technical micro-skills unique to each procedure. These findings indicate that both needle and surgical cricothyrotomy remain viable options for emergency front-of-neck access and suggest that the choice of technique should be prioritized based on practitioner proficiency and equipment accessibility.

## Figures and Tables

**Table 1:** Characteristics of subjects’ level of training in a study comparing procedural completion time and first-pass success of needle and surgical approaches to cricothyrotomy

Level of training, n (%)	All subjects (N = 99)	Needle (n = 51)	Surgical (n = 48)
EM attending	19 (19.2)	11 (21.6)	8 (16.7)
EM resident	68 (68.7)	34 (66.7)	34 (70.8)
Pararescue specialist	12 (12.1)	6 (11.8)	6 (12.5)

*EM*, emergency medicine.

**Table 2:** Comparison of procedural outcome measures for needle and surgical groups in a study comparing procedural completion time and first-pass success of needle and surgical approaches to cricothyrotomy.

	Needle (n = 51)	Surgical (n = 48)
Median completion time, 95% CI (seconds)	56.5, 54–66	65, 59–73
First-pass success, n (%)	48 (94)	45 (94)
Complications, n (%)		
None	37 (73)	32 (65)
Missed or repeated step	7 (16)	-
Not midline	-	8 (19)
Damage to cartilage	-	2 (4)
Damage to trachea	4 (12)	3 (6)
Ectopic catheter or ETT placement	3 (6)	3 (6)

*ETT*, endotracheal tube.
